# Supplementary material for: Research practice, satisfaction, motivation, and challenges among university academics in Kurdistan Region of Iraq
Source: PLoS One. 2024 Apr 25;19(4):e0302540. doi: 10.1371/journal.pone.0302540 (PMC11045054; doi:10.1371/journal.pone.0302540)
Supplement: S1 Appendix — (DOCX) [file pone.0302540.s001.docx]

**S1 Appendix - Questionnaire for assessing the research practice in Kurdistan Region of Iraq.**

1. Name of your university:

2. Name of your college:

3. Name of your department:

4. Your field of specialty:

5. Scientific title

*Select only one*

- Assistant lecturer
- Lecturer
- Assistant professor
- Professor

6. Age (year)

*Select only one*

- Less than 30
- 31- 40
- 41-50
- More than 50

7. How many days per week do you stay in College/Office related to research work?

*Select only one*

- zero
- 1 day
- 2 days
- 3 days
- 4 days
- 5 days

8-How many hours per day do you stay in College/Office/Field related to research work?

*Select only one*

- Zero
- 1 to 2 hours
- 3 to 4 hours
- 5 hours and more

9. What proportion of your working hours is assigned to research?

*Select only one*

- less than 25%
- 25%
- 50%
- 75%
- 100%

10. How many hours per week do you spend on teaching and lecturing?

*Select only one*

- 1-5
- 6-10
- 10-15
- More than 15

11. How many articles do you read per week in your field of interest?

*Select only one*

- None
- 1-2
- 3-4
- 5-6
- More than 6

12. Do you have adequate access to the publications you need to undertake research?

*Select only one*

- Yes
- No

13. If the answer to the previous question is yes, please verify the nature of your access.

*Select only one*

- Personal Subscription
- Institutional/University Subscription
- I only use free articles

14. Do you feel that the research and publishing papers are a part of your job?

*Select only one*

- Yes
- No

15. Are you satisfied with your academic role as a researcher?

*Select only one*

- Yes
- No

16. If you are not satisfied, please mention the reason/s.

*Select only one*

- Poor infrastructure
- No research cores
- No financial motivation
- No academic motivation
- Having a safe academic position without research
- Other

17. What is your main motivation in conducting research?

*Select only one*

- Getting a higher diploma
- Getting academic promotion
- Job responsibility
- Passion for science and continuous learning

18. Have you ever participated in any workshop for writing a research proposal in support of a grant application?

*Select only one*

- Yes
- No

19. Have you ever applied for any international grants for your research?

*Select only one*

- Yes
- No

20. If the answer to the previous question is yes, please list them.

21. Number of published research papers inside the country (Iraqi journals).

*Select only one*

None

- 1-5
- 5-10
- 10-15
- More than 15

22. Number of published research papers outside the country (International journals).

*Select only one*

- None
- 1-5
- 5-10
- 10-15
- More than 15

23. Do you think the Ministry of Higher Education and Scientific Research has a sufficient vision, mission, and plan for conducting effective research by academic staff?

*Select only one*

- Yes
- No
- Somehow

24. Could you list the ministries or institutions to which your research is related, as well as any private sector entities?

25. Do you think your research results were used by related local authorities to improve or solve any problems in Kurdistan?

*Select only one*

- Yes
- No

26. Have you shared your research findings with the relevant ministries or institutions?

*Select only one*

- Yes
- No

27. If yes to the previous question, how did you share your research findings?

*Check all that apply.*

- Sharing the paper or journal
- Sharing a summary in Kurdish language
- Presentation (seminar)
- Workshop
- Conference presentation
- Planning for a project
- other

28. What is/are the main challenge/s facing conducting effective research?

*Select only one*

- Lack of Funding
- Lack of research infrastructure and technology
- Lack of industry-academia cooperation
- No support and encouragement
- Other

29. Do you think that the research conducted by Kurdistan academic scholars is effective and produces new knowledge that is recognized worldwide?

*Select only one*

- Yes
- No

30. Do you have any research collaborations in your research, either locally or internationally?

*Select only one*

- Yes
- No
- Previously I had

Thank you for your valuable contribution.
