# Supplementary material for: Research practice, satisfaction, motivation, and challenges among university academics in Kurdistan Region of Iraq
Source: PLoS One. 2024 Apr 25;19(4):e0302540. doi: 10.1371/journal.pone.0302540 (PMC11045054; doi:10.1371/journal.pone.0302540)
Supplement: Attachment — Submitted filename: Response to Reviewers.pdf [file pone.0302540.s002.pdf]

PLOS One

Subject: Resubmission of revised manuscript (PONE-D-24-10517)

Research practice, satisfaction, motivation, and challenges among university academics in Kurdistan Region of Iraq

Dear Editor,
Thank you very much for sharing the reviewers' comments and suggestions on our manuscript. We have found them very helpful in improving the quality and clarity of the manuscript.

We have made the necessary revisions by responding to the suggested comments. Please find below explanations of the revision made through point-to-point responses to the comments. All the changes and corrections are highlighted in the manuscript.

Thank you very much for considering this revised version of the manuscript.

Sincerely,

**Reviewer 1**	
In general, the paper is well-written and easy to follow. The authors highlight an issue that is of real concern. Thus, I admire their initiative.<br><br>There are some minor points that need to be addressed, as follows.	Thank you very much for the positive comments.<br><br>Thank you for the comments and suggestions that we found very useful.
**General notes:**<br>1. I believe one of the factors that affects research productivity is access to scientific journals and books. In the Kurdistan region, the majority of universities, public ones in particular, do not provide electronic access to their academic staff. This is something that, I believe, has to be discussed in the manuscript.	Thank you very much for this valuable comment. The issue of access to scientific journals and books is now thoroughly discussed in the Discussion section (Pages 12-13, lines 211-224). It is also included in the Conclusions (Page 18, lines 346-347)
2. There is financial support for publication by some universities (some public and the majority of private universities). Again, this has to be explored.	The issue of financial support for research, the support by some universities, and related issues are now discussed in detail in the Discussion section (Page 14, lines 260-264).
3. The authors have to mention the rules by the Ministry of Higher Education in KRG as these rules are amended frequently and affect the quality of higher education in KRG.	The ministry's main rules and the effect of their frequent amendments on higher education are now discussed in detail (Page 16, lines 291-299).

4. There needs to be a clearer collaboration between the Ministry of Higher Education and the Ministry of Planning in KRG. This discourages academic staff from doing research, as they believe that policymakers in the KRG do not hear their voices. Thus, it has to be added to the discussion section.	Thanks for this comment. We have now discussed the issue of collaboration and knowledge transfer in detail in the Discussion section (Page 16, lines 307-312). It is also included in the Conclusions (Page 18, lines 347, 355-356).
5. The lack of a clear rule for academic staff with no publication and an old style of academic title promotion (the latest rule is from 2016 in KRG) could also be related to the fact that research publication is not taken seriously by the academic staff. Again, this has to be explored.	This lack of clear rules for academic staff with no publication is now discussed in detail in the Discussion section (Page 15, lines 281-288). It is also included in the Conclusion (Page 18, lines 352-354)
6. The salary issue should also be mentioned, as government employees in KRG suffer delays in their monthly salary KRG government.	The issue of delays in staff salary is now discussed in the Discussion section (Page 14-15, lines 264-268).
**Specific notes**:<br>1. A copy of the questionnaire has to be added as a figure in the method section.	A copy of the questionnaire is now provided as the supporting information file (Page 5, lines 118-119 and S1 Appendix file).
2. Is the questionnaire validated? This has to be stated in the methodology.	Details are provided about the validation of the questionnaire (Page 6, lines 120-123).
3. The ethical committee's number and approval date must be added.	The ethical committee approval number and date are provided (Page 6, line 137).
4. The methods sections should be sectioned as follows: study design, questionnaire, participants, and statistical analysis.	The Methods section is now divided to sub-sections, as advised (Page 5-6, lines 109, 112, 127, and 131).
5. What are the study's limitations? This has to be added to the discussion. There are inherent issues with the questionnaire study.	The study limitations are now provided and discussed in detail at the end of the Discussion section (Pages 17-18, lines 333-343).
6. Table 1 better to be presented as a pie chart.	Table 1 is changed to a pie chart (Page 7, line 145 and Figure 1).
